# Validation of SYBR green I based closed tube loop mediated isothermal amplification (LAMP) assay and simplified direct-blood-lysis (DBL)-LAMP assay for diagnosis of visceral leishmaniasis (VL)

**DOI:** 10.1371/journal.pntd.0006922

**Published:** 2018-11-15

**Authors:** Keerti Kaumudee Dixit, Sandeep Verma, Om Prakash Singh, Dharmendra Singh, Akhil Pratap Singh, Ratan Gupta, Narendra Singh Negi, Pradeep Das, Shyam Sundar, Ruchi Singh, Poonam Salotra

**Affiliations:** 1 ICMR-National Institute of Pathology, Safdarjung Hospital Campus, New Delhi, India; 2 Faculty of Health and Biological Sciences, Symbiosis International (Deemed University), Pune, India; 3 Department of Medicine, Institute of Medical Sciences, Banaras Hindu University, Varanasi, Uttar Pradesh, India; 4 Rajendra Memorial Research Institute of Medical Sciences (RMRIMS), Patna, India; 5 Department of Paediatrics, Safdarjung Hospital and Vardhman Mahavir Medical college, New Delhi, India; 6 Department of Medicine, Safdarjung Hospital and Vardhman Mahavir Medical College, New Delhi, India; US Food and Drug Administration, UNITED STATES

## Abstract

**Background:**

The World Health Organization has targeted elimination of visceral leishmaniasis (VL) in the Indian subcontinent (ISC) by 2020. Despite distinctive decline seen in the number of VL cases in ISC, there is still a quest for development of a diagnostic test which has the utility for detection of active infection and relapse cases and as a test of cure. The present study validated the sensitivity and specificity of SYBR Green I based closed tube LAMP assay reported by us for diagnosis of VL.

**Methodology:**

The validation study was carried out at two endemic sites in India, located at Rajendra Memorial Research Institute of Medical Sciences (RMRIMS), Patna and Institute of Medical Sciences (IMS), Banaras Hindu University (BHU), Varanasi. Standard operating protocols were provided at the two sites for applying LAMP assay on confirmed VL cases. The diagnostic accuracy of LAMP assay was evaluated by Receiver operator curve (ROC) analysis. Furthermore, a simplified LAMP assay based on direct blood lysis, DBL-LAMP, was developed and verified for its diagnostic accuracy.

**Principal findings:**

A total of 267 eligible participants were included in the study which comprised of 179 VL cases and 88 controls. Sensitivity and specificity of the LAMP assay were 98.32% (95% C.I– 95.2–99.7%) and 96.59% (95% C.I.-90.4–99.3%), respectively. ROC curve analysis depicted no significant difference between area under curve (AUC_ROC_) for LAMP assay and rK39 RDT, indicative of LAMP as an excellent diagnostic test. DBL-LAMP assay, performed on 67 VL and 100 control samples, yielded a sensitivity of 93.05% (95% C.I- 84.75–97%) and specificity of 100% (95% C.I.- 96.30–100%).

**Conclusions/Significance:**

The validated closed tube LAMP for diagnosis of VL will provide impetus to the ongoing VL elimination programme in ISC. The assay based on direct blood lysis promotes its scope for application in field settings by further reducing time and cost.

## Author summary

Definitive diagnosis of visceral leishmaniasis(VL) by demonstration of amastigotes by microscopy is invasive and risky. Serology based diagnosis using rK39 rapid diagnostic test(RDT) has excellent sensitivity of~97% when combined with clinical symptoms but is inconclusive for detection of active infection and relapses due to persistence of anti-leishmanial antibodies. The developed SYBR Green I based closed tube LAMP assay overcomes these constraints and further, direct blood lysis (DBL)-LAMP, makes it more suitable for field application. The study involved validation of LAMP assay at two endemic sites in India, on a total of 179 VL patients confirmed by rK39 RDT and/ or microscopy and 88 controls. The assay was highly sensitive (98.32%) and specific (96.59%). Further, DBL-LAMP assay yielded a sensitivity and specificity of 93.05% and 100%, respectively. In conclusion, the study has validated the field potential of LAMP assay for diagnosis of VL which will provide momentum to ongoing VL elimination in the Indian subcontinent (ISC).

## Introduction

Visceral leishmaniasis (VL) is one of the most neglected infectious diseases with an annual incidence of 50 000 to 90 000 new cases worldwide [1]. Cases of VL are characterized by irregular bouts of fever, weight loss, hepatosplenomegaly, hypergammaglobulinemia, pancytopenia and anaemia. Post kala-azar dermal leishmaniasis (PKDL), a dermatotropic form of VL which is characterised by macular, maculopapular and nodular lesions in a patient who has been apparently cured of VL, are presumed to pay a crucial role in maintaining parasite reservoir especially during interepidemic periods of VL.

Case management, control and surveillance of a disease rely on definitive diagnosis. It has been reported that shortening the time from health care seeking to diagnosis could bring about substantial reduction in incidence of VL in endemic areas of the Indian subcontinent (ISC) [2]. Dramatic decline of >3 fold has been reported in number of VL cases in 54 districts endemic for VL in India (20600 in 2012 to 5758 cases in 2017) [3]. On the basis of number of reported cases and the population at risk the calculated prevalence for VL is 0.0035%.

Diagnosis of VL in ISC is based on combining the clinical examination involving a history of fever of more than 2 weeks with splenomegaly and hepatomegaly along with positive parasitological or serological tests [1, 4]. Demonstration of amastigotes by microscopy is invasive, risky and technically demanding with the limitation of low sensitivity [2, 4–6]. Serology based tests such as indirect immunofluorescence antibody test(IFAT), enzyme linked immunosorbent assay(ELISA), recombinant antigen-based immunochromatography test(ICT) and western blot have limitations in terms of cross reactions in the presence of other diseases and inability to differentiate between active and past infections. Direct agglutination test (DAT), a semi-quantitative method based on the visual agglutinations [7], depicts high sensitivity (97.1%) and specificity (95.7%) [2], and has been extensively validated but is limited by its complex procedure and antigen variability [8–11]. The rK39 antigen rapid diagnostic test (RDT) has played a pivotal role in VL elimination and is in accordance with all the affordable, sensitive, specific, user-friendly, rapid and robust, equipment-free and deliverable (ASSURED) criteria [12]. It depicts distinctive sensitivity (97%) and specificity (90.5%) [2] in diagnosis of VL and based on WHO’s recommendation the use of rk39 RDT has been adopted by the national VL elimination programmes in India, Bangladesh and Nepal for diagnosis of VL. VL surveillance under national programme involves two types, active and passive surveillance [13]. rK39 RDT demonstrates remarkable sensitivity and specificity, however is not adequate for detection of active infection and has limited utility as a marker for disease progression and relapse, nor can it be used as a test of cure.

Molecular methods employing polymerase chain reaction (PCR) based methods overcome these constraints along with provision of much higher sensitivity and specificity. Numerous PCR procedures [14–19] have been employed for diagnosis of leishmaniasis including triplex PCR [20], multiplex PCR [21], restriction fragment length polymorphism analysis and nested PCR [22]. Quantitative PCR (Q-PCR) is an exceptionally sensitive and specific assay for diagnosis of VL that enables rapid and accurate quantification of parasite burden [23].However, these molecular methods pose issues in field applicability as they require well equipped laboratory, expensive instruments and reagents for complicated post-PCR analysis.

Loop mediated isothermal amplification (LAMP) has evolved as an efficacious tool in diagnostics [24–27]. The attributes of LAMP include isothermal amplification owing to strand displacement property of *Bst* polymerases [24], high specificity by the use of 6 set of primers targeting 8 regions,10^9^−10^10^ times amplification efficiency within 15–60 minutes of incubation, generation of enormous amplified product enabling naked eye visual detection of positives[28–30].Additionally, it demonstrates high tolerance towards inhibitory components present in DNA samples[26,31]and can be used directly with crude unprocessed clinical samples without any inhibition seen during amplification process as in Q-PCR. The assay has been successfully employed for detection of *Plasmodium* sp. [32, 33], *Toxoplasma gondii* [34], *Schistosoma japonicum*[35], *Trypanosoma*[36,37], *Cryptosporidium*[38], *Giardia*[39] etc.

Numerous studies have successfully employed LAMP assay in diagnosis of leishmaniasis [30, 40–50].However, some of the studies reported so far have shown that despite all the advantages, LAMP assay is invariably prone to aerosol contamination which poses a stumbling block to its field application. Nonetheless, our closed tube LAMP assay overcomes this hitch by application of SYBR Green I on the inner side of the lid of the tube [50]. The approach abolishes the need to open the tube at the end of the reaction which often leads to false positives. Recently, calcein based Loopamp® *Leishmania* detection kit has been developed by the Eiken Chemical Co., FIND and partners, which has demonstrated appreciable diagnostic performance [51,52].

In an attempt to further enhance the potential of LAMP assay in diagnosis, the use of direct crude clinical sample instead of extracted DNA has been attempted for diagnosis of many parasites such as *Leishmania*, *Trypanosoma* and *Plasmodium* [47,53–55]. On similar lines, we have employed simplified direct blood assay for diagnosis of VL that eliminates the step of DNA isolation making it both time and cost efficient.

Here, we attempted to validate, our closed tube LAMP assay that has not only depicted distinctive sensitivity (96.9%) and specificity (100%) for diagnosis of VL but also has shown exemplary sensitivity (97%) and specificity (100%) for detection of PKDL [50]. To the best of our knowledge, this is the first study to assess and validate the diagnostic accuracy of LAMP assay for mass surveillance of leishmaniasis in VL endemic regions of ISC. Further, the study has explored the application of LAMP assay using direct blood lysis, eliminating the DNA isolation step for diagnosis of VL.

## Methods

### Ethics statement

The recruitment of patients complies with principles laid down in the Helsinki declaration 1975 and later revised in Edinburgh, 2000 on human rights. The study was approved by and carried out under the guidelines of the Ethical Committee of Safdarjung Hospital, Vardhman Mahavir Medical College, New Delhi, India. All patients or responsible adults provided written informed consent for the collection of samples and subsequent analysis. The study for validation of LAMP assay was approved and carried out under the guidelines of the Ethical Review Committee of ICMR-Rajendra Memorial Research Institute of Medical Science (RMRIMS), Patna, India and Ethical Review Board of Banaras Hindu University (BHU), Varanasi, India. Written informed consent was obtained from all the patients included in the study.

### Parasite DNA

*L*. *donovani* AG83 (MHOM/IN/83/AG83) was used as a positive control for the assay. The parasites, cultivated in Medium 199 supplemented with 25 mM HEPES pH 7.5 and 10% fetal calf serum, were harvested in late log phase and washed in phosphate buffered saline. DNA was isolated using Promega genomic wizard DNA isolation kit (Promega, Madison, WI, USA) as per the manufacturer's instructions.

### Sample size calculation for Validation study

For validation of LAMP assay; a minimum sample size of 45 VL cases was calculated based on mean sensitivity of 97% for assessment of the accuracy of the assay as per the previous published data [50]. The specificity of the study was 100%, thus a minimum of 39 non-infected controls was estimated to be tested for a confidence level of 95%.

$N=\frac{Z_{\alpha /2}{}^{2}\;P(1‑P)}{d^{2}}$ where, P is the sensitivity or specificity ascertained by the previous published data or clinician’s experience/judgment and for α = 0.05, Z_α/2_is 1.96. d = precision of estimate (i.e. maximum marginal error [56].

Sample size calculation can also be done on the basis of VL prevalence. Considering the reported total number of VL cases as 5758 with a population of 165.4 million at risk in 54 districts endemic for VL in 4 states in India in year 2017 [3], the estimated prevalence of VL in India is 0.0035%. The sample size for validation study was calculated using **Buderer's formula** for an anticipated specificity of 95% [56, 57]. A total of 73 cases need to be included in the study to reach a precision level of 5% with value of α = 0.05.

### Validation study sites and clinical samples

Validation of the LAMP assay was conducted (during 2013 to 2016) at two sites–Rajendra Memorial Research Institute of Medical Sciences (RMRIMS), Patna, Bihar, India and Institute of Medical Sciences (IMS), Banaras Hindu University (BHU), Varanasi, India.

VL suspects (having fever for more than 2 weeks and coming from VL endemic area) were tested using the rapid diagnostic test (rK39 strip test, InBios, India) and/or microscopic examination of Giemsa stained splenic or bone marrow aspirates for the presence of *L*. *donovani* amastigotes. Venous blood was collected in heparinized tubes from all samples. A total of 267 cases were a part of this large-scale validation study which included 179 confirmed VL patients (VL group) and 88 control samples (Non-VL group) consisting of healthy and endemic healthy volunteers. The patients reporting at IMS, BHU (n = 129) [62.7% males and 37.2% females; median age 21years (range-7-62 years)] and at RMRIMS (n = 50) [36% males and 64% females; median age 23years (range-4-60 years)] were included at the pre-treatment stage. Blood samples from healthy volunteers at BHU (n = 53) [67.9% males and 32.07% females; median age 40 years (range-11-65 years)] and at RMRIMS (n = 35) [57.14% males and 42.8% females; median age 29 years (range-8-65 years)] were included for determining the specificity of the LAMP assay. Patients seropositive for human immunodeficiency virus, hepatitis B and C, tuberculosis or suffering from any other systemic ailments were excluded from the study. No cases of co-morbidity were included. Pregnant or lactating women were also excluded from the study. The study was conducted in accordance with the criteria laid out by the standard for the reporting of diagnostic accuracy studies (STARD) (**S1 Appendix; S2 Appendix**)[58].

### Clinical samples for direct blood lysis (DBL)-LAMP

VL patients originating from Bihar and reporting at Department of Medicine, Safdarjung Hospital, Vardhman Mahavir Medical College (VMMC), New Delhi were included in the study at pre-treatment stage. The patients presenting with typical symptoms of VL as irregular bouts of prolonged fever, splenomegaly, hepatomegaly, leucopenia and weight loss who were rk39 RDT positive were included in this study. All the cases were confirmed by Q-PCR assay [23]. Venous blood was collected from VL patients (n = 17). Blood samples from healthy volunteers (n = 30), malaria patients (n = 25) and typhoid patients (n = 25) were collected as control. Further, DBL-LAMP assay was also performed on VL blood samples (n = 55) and endemic healthy controls (n = 20) samples at IMS, BHU.

### Quality assurance and quality control

Research scholars at both validation sites RMRIMS and IMS, BHU underwent training program organised by NIP for performing LAMP assay for diagnosis of VL. Standard operating protocols (SOPs) were prepared and provided by NIP at the two validation sites (**S3 Appendix)**. For assuring accuracy and precision of LAMP assay, all the reagents needed for performing the assay were provided at both validation sites. The LAMP assay was performed in batches of 10 reactions each time, inclusive of 8 confirmed VL cases along with a positive (1ng/μl) and a negative control.

### DNA isolation from clinical samples

Blood was collected in heparinized tubes. DNA extraction was done using QIAmp DNA Blood mini kit (QIAGEN, Hilden, Germany) according to manufacturer's instructions. DNA was isolated from 200 μl of blood and eluted in 50 μl of nuclease free water.

### LAMP assay

**Fig 1:**
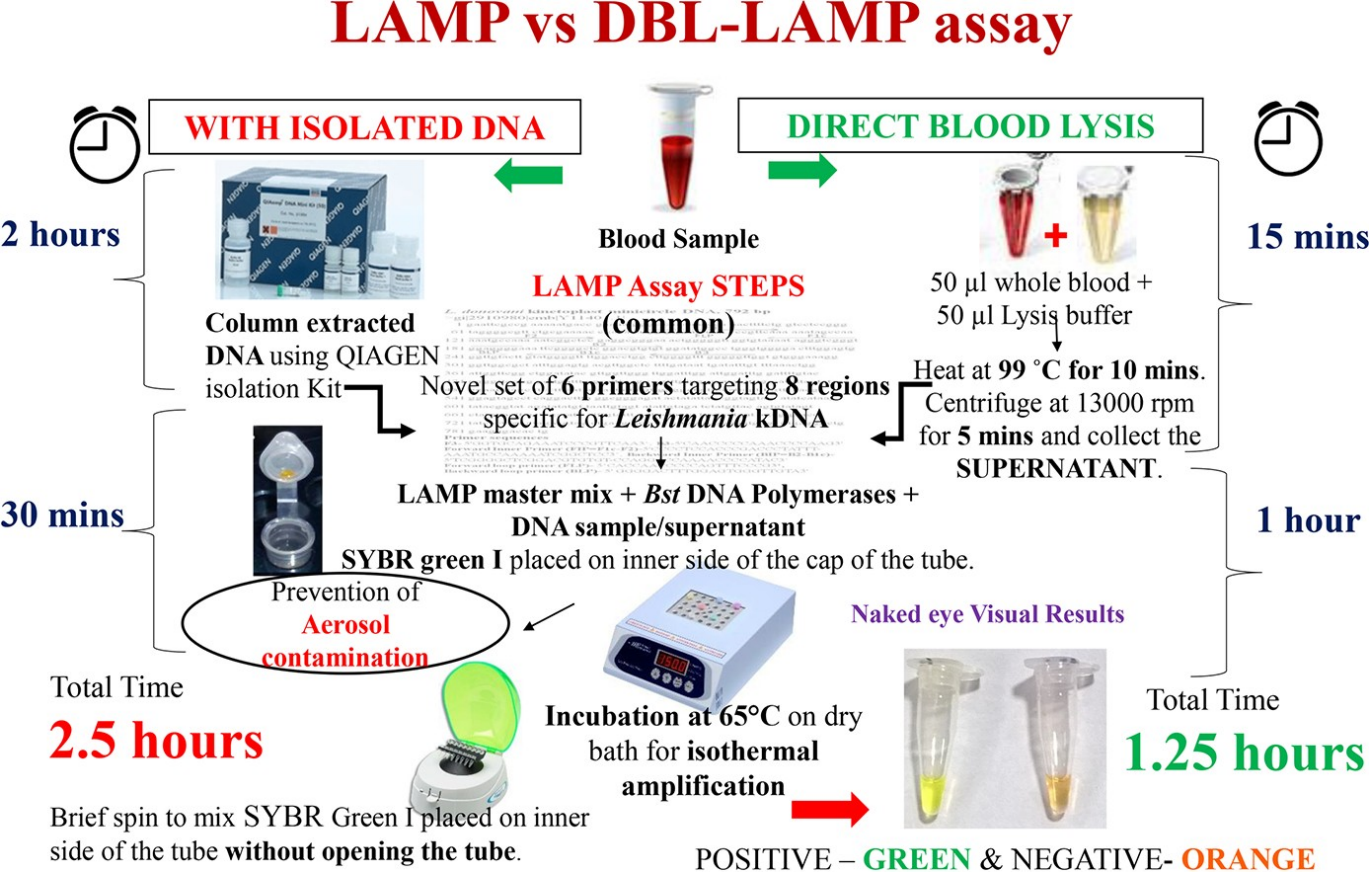
Comparative standard operating protocol of LAMP assay (performed with column extracted DNA) vs. direct blood lysis (DBL)-LAMP assay (performed with direct blood lysed supernatant).

The closed tube LAMP assay was performed as described earlier [55].The reaction was performed in 25 μl of reaction mixture containing 40 pmol each of FIP and BIP primers, 5 pmol each of F3 and B3 primers,20 pmol each of the FLP and BLP **(S4 Appendix)]**, 1.4 mM of each deoxynucleoside triphosphate, 0.8 M betaine, 20 mM Tris-HCl, pH 8.8, 10 mM KCl, 10 mM (NH_4_)_2_SO_4_, 8 mM MgSO_4_, 0.1% TritonX-100, 8 units of *Bst* DNA polymerase large Fragment (New England Biolabs, Ipswich, MA, USA), and 2 μl of DNA sample. 1 μl of 1:10 diluted SYBR green I (Molecular Probes, Eugene, OR, USA) was placed on the inner side of the tube. The closed tube was then incubated on a dry bath at 65°C for 30 minutes. At the end of reaction, the tubes were allowed to cool down to room temperature and a brief spin was given to allow mixing of SYBR Green I with the amplified product. The positives instantaneously turned green while the negatives remained orange (**Fig 1**).

### DBL-LAMP assay

Equal volume of heparinized blood was mixed with mammalian cell lysis buffer (Gold Biotechnology, USA). 50μL of blood was mixed with 50μL of lysis buffer. The mix was incubated at 99°C on a dry bath for 10 minutes and centrifuged at 13,000 rpm for 5 minutes. 5μl of the supernatant was used for performing DBL-LAMP assay and the remaining supernatant was stored at -20°C until further use. The LAMP reaction was performed using the reaction mixture as described above with 5μl of supernatant as template DNA. The incubation time at 65°C was increased to 60 minutes. The positive samples turned instantly green on giving a brief spin at the end of 60 minutes whereas negatives remained unchanged.

### Statistical analysis

Statistical analysis was carried out using MedCalc statistical software (version 16.8.4). Receiver operating curves (ROCs) were used to analyze the diagnostic accuracy of the tests [59]. The area under ROC curves (AUC_ROC_) were compared as described by Hanley and McNeil [60]. A test was considered significant if p value was less than 0.05.

## Results

### Performance of the LAMP assay for diagnosis of VL at the validation sites

**Fig 2:**
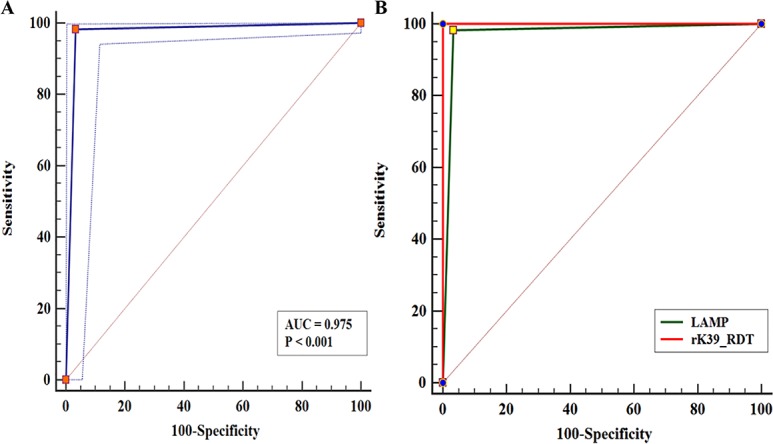
Receiver operating curve (ROC) curve analysis for assessing the diagnostic accuracy of LAMP assay. **(A) ROC curve for LAMP assay for diagnosis of VL at validation sites.** The AUC_ROC_ for LAMP assay was 0.975; p <0.0001 depicting it to be an excellent diagnostic test **(B)-ROC curve analysis showing comparison of rK39 RDT and LAMP.** The AUC_ROC_s for rK39 RDT and LAMP were 1.00 and 0.975 respectively. No remarkable difference between two AUC_ROC_s 0.0254 (SE, 0.0125, p -0.1053).

Parasite DNA was detectable in 176 of 179 VL cases tested for validation of LAMP assay at the two centres, giving it a sensitivity of 98.32% [95% C.I. - 95.2–99.7%]. The assay was negative in 85 out of 88 endemic healthy control samples, giving it a specificity of 96.59% [95% C.I. - 90.4–99.3%] (**Table 1**). ROC curve, drawn for evaluating the diagnostic accuracy of the assay, revealed that the LAMP assay was able to discriminate between VL and healthy controls with an AUC_ROC_ of 0.975; p < 0.0001 and Youden index J of 0.9491, indicative of an excellent diagnostic test (**Fig 2A**). Furthermore, comparative ROC curves were generated for assessment of the clinical value of LAMP assay index test with rK39 RDT taken as reference test for diagnosis of VL. The comparative results revealed insignificant difference between the AUC_ROC_ of the two diagnostic tests with values of 0.975 and 1.00 for LAMP assay and rK39 RDT, respectively (z-statistic– 2.03) (**Fig 2B**). Site wise analysis at RMRIMS and IMS, BHU in validating LAMP assay for diagnosis of VL has been summarised in **Table 1.(S5 Appendix)** provides the cross-tabulation of the index test results (or their distribution) by the results of the reference standard.

**Table 1:** Sensitivity and specificity of LAMP assay for diagnosis of VL at the two validation sites.

Validation Site	Sample	Cases tested	Cases positive	Sensitivity/ Specificity (95%C.I.)
**IMS, BHU, Varanasi, India**	**VL**	**129**	**127**	**98.45%** (94.5–99.8%)
	**Controls**	**53**	**2**	**96.23%** (87.00–99.5%)
**RMRIMS, Patna, India**	**VL**	**50**	**49**	**98%** (89.4–99.9%)
	**Controls**	**35**	**1**	**97.14%** (85.1–99.9%)
**Combined**	**VL**	**179**	**176**	**98.32%** (95.2–99.7%)
	**Controls**	**88**	**3**	**96.59%** (90.4–99.3%)

**Fig 3:**
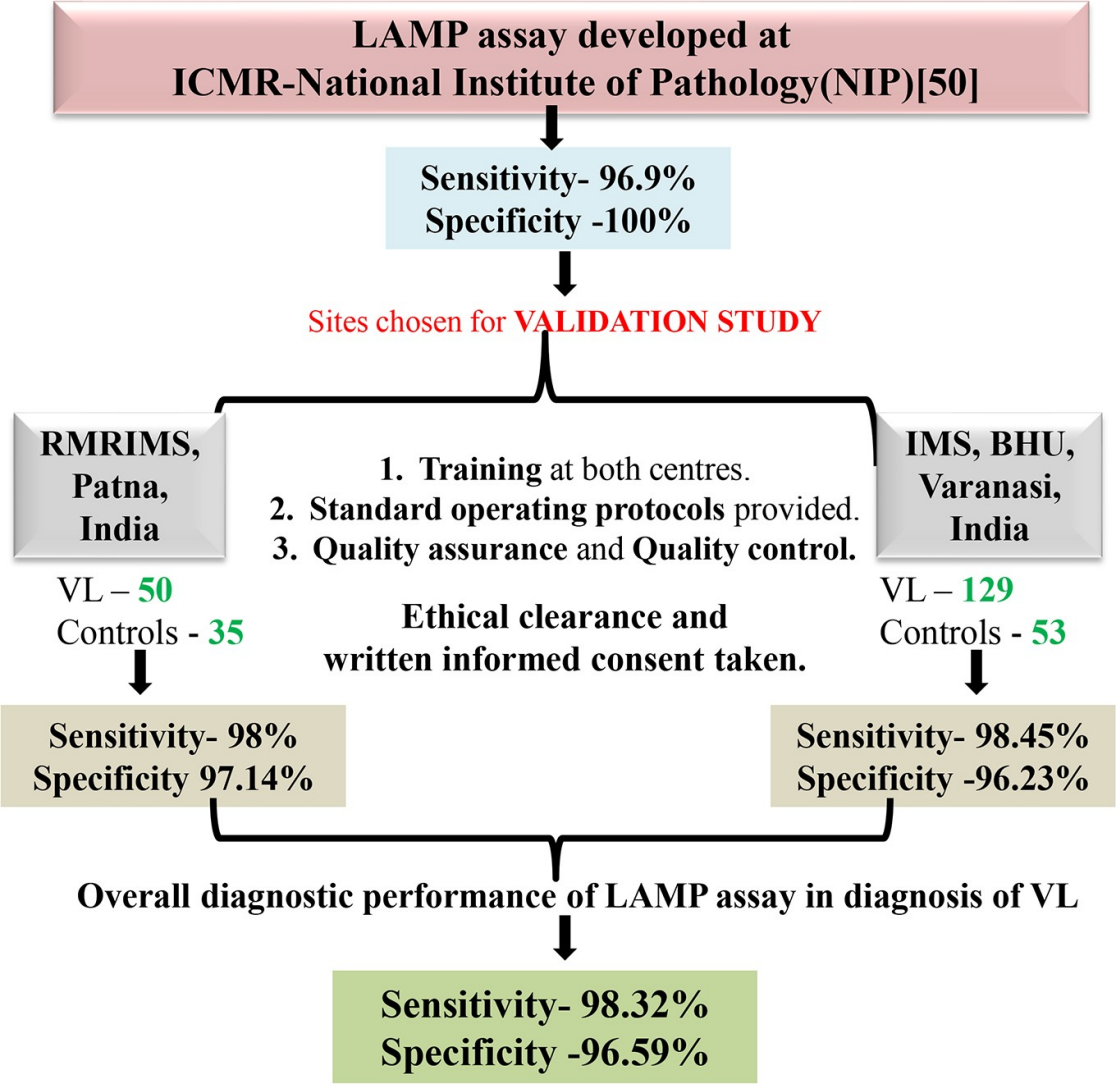
Schematic representation of procedure and results obtained for validation study of LAMP assay at RMRIMS, Patna, India and IMS, BHU, Varanasi, India. Sensitivity and specificity of DBL-LAMP assay for diagnosis of VL.

The results of diagnostic performance of the closed tube LAMP assay at the two validation sites, RMRIMS and IMS, BHU, corroborated with reported sensitivity and specificity of the LAMP assay for diagnosis of VL observed at NIP **(Fig 3).**

**Fig 4:**
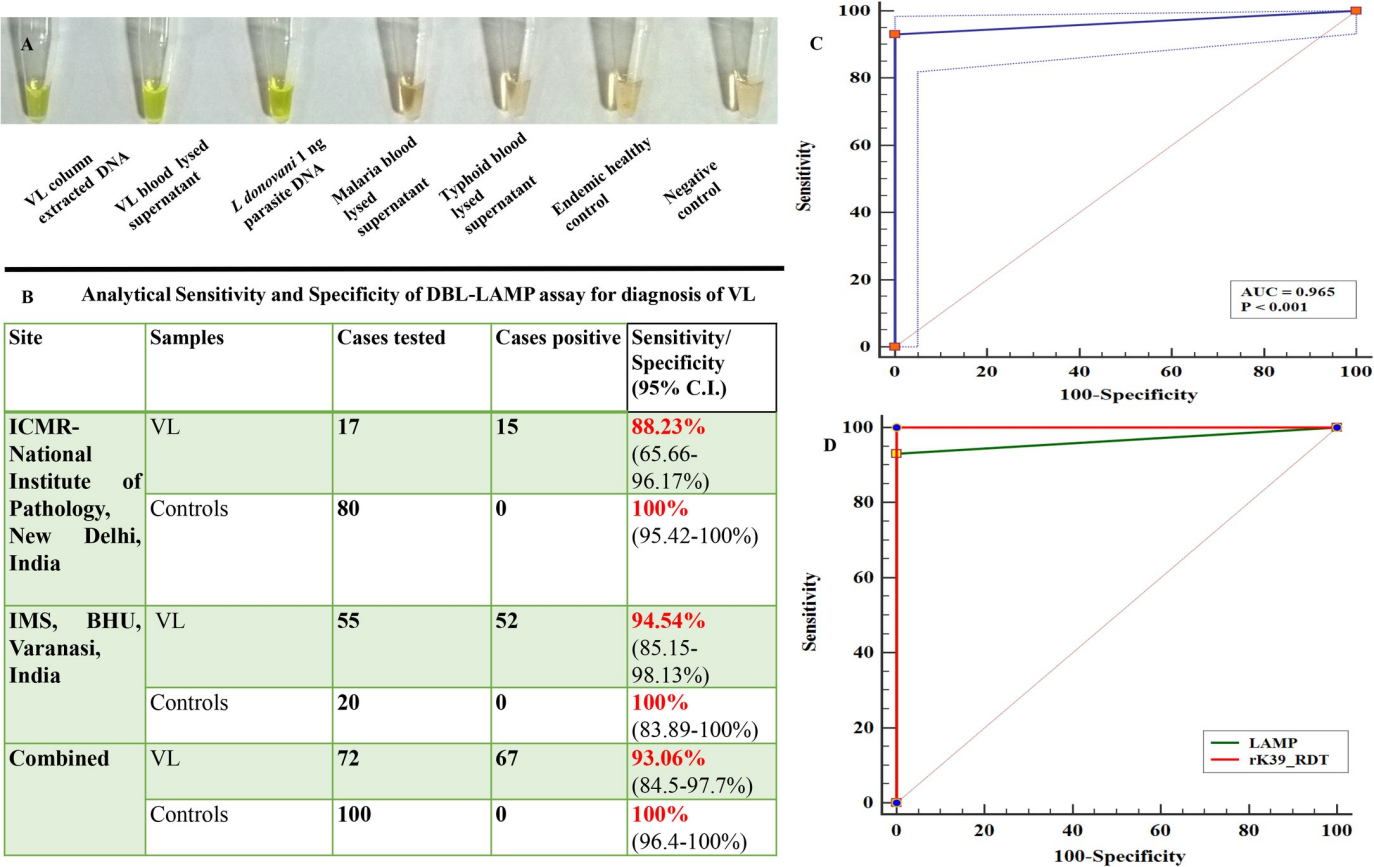
Simplified Direct Blood Lysis (DBL)-LAMP assay for diagnosis of VL. **(A)- Visual appearance of amplified product on addition of diluted SYBR Green I for LAMP and DBL-LAMP assay at the end of reaction**. The DBL-LAMP assay was performed using direct blood lysed supernatant instead of column extracted DNA; as described in the methods section. Negative samples remained orange whereas positives turned instantly green on giving brief spin allowing mixing of SYBR Green I (1:10 diluted) at the end of the reaction. **(B)- Analytical sensitivity and specificity of DBL-LAMP assay for diagnosis of VL. (C)-ROC curve for DBL-LAMP assay for diagnosis of VL.** The AUC_ROC_ for DBL-LAMP assay was 0.965; p<0.0001 indicative of an excellent diagnostic test. **(D)- Comparative ROC curve analysis for DBL-LAMP assay vs rK39 RDT.** The AUC_ROCs_ for rK39 RDT and DBL-LAMP were 1.00 and 0.965 respectively. No remarkable difference between the AUC_ROC_s 0.0347(SE 0.0176, p-1.971).

The direct blood lysis (DBL)-LAMP assay, was performed on confirmed VL cases and controls. The assay was established for diagnosis of VL at NIP where it was positive in 15 out of 17 cases giving a sensitivity of 88.23% [95%C.I. - 65.55–96.17%]. The assay was negative for all 80 control blood samples inclusive of malaria, typhoid and healthy controls, giving it a specificity of 100% [95% C.I.-95.42–100%](**Fig 4A**). Further, the assay was applied on confirmed VL samples at IMS, BHU where the DBL-LAMP assay was able to detect parasite DNA in 52 out of 55 VL cases. The assay was negative for all 20 endemic healthy control samples. Overall, the DBL-LAMP assay was positive in 67 out of 72 direct blood lysed supernatant of VL patients giving it a sensitivity of 93.06% [95% C.I- 84.5–97.7%]. The assay was negative for all the 100 control blood samples tested at the two centres, giving it a specificity of 100% [95%C.I.-96.4–100%] (**Fig 4B)**. ROC curve drawn to assess the accuracy of assay for diagnosis of VL revealed that DBL-LAMP was able to discriminate between VL and controls with an AUC_ROC_ of 0.965; p<0.0001and Youden index J of 0.9306 indicative of an excellent diagnostic test **(Fig 4C)**. The comparative ROC curve analysis was also done to assess diagnostic accuracy of DBL-LAMP assay with that of rK39 RDT. The analysis showed remarkable concordance between the AUC_ROC_ of the two tests with values of 1.00 and 0.965 for rK39 RDT and DBL-LAMP assay respectively(Z-statistic –1.971) (**Fig 4D)**.

## Discussion

Timely diagnosis of a disease is critical to its case management, control and surveillance. The goal of VL elimination programme in ISC is to achieve annual incidence of <1 VL case per 10,000 inhabitants for 3 consecutive years at upazila, administrative block and district levels in Bangladesh, India and Nepal, respectively. In spite of impeccable progress of declining trend in VL cases, 90 of the 456 blocks (20%) still remain endemic for VL in India [61]. Also, recent reports of sporadic cases of VL from new ecological niches in India, Nepal and Bhutan are a matter of grave concern in terms of expansion of VL in non-endemic regions [62–67].

Recent studies on transmission models of VL in ISC have concluded that *L*. *donovani* transmission will continue even after 2020 and thus the continued intervention which includes active case detection should remain in place until the breaking of transmission cycle of VL is achieved [68]. Further, potential hurdle posed by PKDL cases and asymptomatics, makes it challenging in sustaining the VL elimination goal. However, rk39 RDT is not adequate for detecting PKDL and asymptomatics. Diagnostic tests of high specificity, even with moderate sensitivity, that are applicable to detect early stage of active infection, would be suitable for control of VL and prevention of its resurgence [2].

The present study focussed at validating the established LAMP assay for diagnosis of VL. The validation study was specifically conducted at sites endemic for VL in India. This large-scale validation study depicted exemplary sensitivity of 98.32% [95% C.I. 95.2–99.7%] and specificity of 96.59% [95% C.I.-90.4–99.3%] at both the sites, which was in congruence with our reported sensitivity of 96.9% [95%C.I.- 89.6–99.2%] and specificity of 100% [95% C.I.– 96.3–100%] [50]. The false negative result of 3 cases of VL (1 at RMRIMS and 2 at IMS, BHU) by LAMP assay may be due to low parasitaemia in blood, though we failed to confirm the same due to lack of Q-PCR data at the validation sites. Efforts were undertaken to longitudinally follow up the 3 endemic healthy controls who gave positive results in the LAMP assay (1 at RMRIMS and 2 at IMS, BHU). The one at RMRIMS returned with symptoms of VL infection on follow up while the other two at IMS, BHU were lost to follow up. The ROC curve analysis for assessing the diagnostic accuracy of LAMP assay for diagnosis of VL gave an AUC_ROC_ of 0.975, making it an excellent diagnostic test. The comparative ROC curve analysis of LAMP assay with rK39 RDT, at both the sites demonstrated concordance between the AUC_ROC_ that is indicative of superlative and comparable diagnostic accuracy of LAMP assay.

The DBL-LAMP assay for VL diagnosis was easier to perform along with reduction in cost and turnaround time, putting it one step forward towards field application. The overall time for performing LAMP assay was reduced to half i.e. 1.25 hours when direct blood lysis was used rather than column extracted DNA which surpassed the requirement of DNA isolation by the use of crude direct lysed supernatant in place of DNA. After initial establishment of DBL-LAMP in diagnosis of VL at NIP, the assay was verified on confirmed VL cases and controls obtained from IMS, BHU. The assay gave a sensitivity of 94.54% [95% C.I-85.15–98.13%] and specificity of 100% [95%C.I-83.89–100%]. The ROC curve analysis of DBL-LAMP assay gave an AUC_ROC_ of 0.965, proving it to be an excellent diagnostic test.

Regardless of successful development and validation of the LAMP assay, there were certain limitations of the study that included lack of Q-PCR data for VL cases and loss to follow up of the two endemic healthy controls that tested positive in LAMP assay at IMS, BHU. Also, the positive predictive values (PPVs), negative predictive values (NPVs) and accuracy have not been discussed since the prevalence of VL as per the sample size used is not the true indication of actual VL prevalence in India.

Further, identifying cases of PKDL, VL-HIV co-infection and asymptomatics harbouring *Leishmania* is critical for sustaining VL elimination. The studies evaluating the available methods for diagnosis of HIV-*Leishmania* co-infection have established PCR and Q-PCR as effective tools [69, 70]. Recently, cent percent sensitivity has been reported in detection of VL-HIV co-infection using LAMP assay [49]. Moreover, as VL transmission in ISC is presumed to be anthroponotic, detection and treatment of PKDL needs to be an indispensable component of VL elimination programme. The studies pertaining to application of LAMP and DBL-LAMP assay in detection of PKDL and asymptomatics are underway in our lab. To support the regional VL elimination initiative, a simple, yet highly sensitive (>95%), specific (>90%) and reproducible diagnostic test that can be deployed in field settings is required [71]. The present study advocates the field utility of LAMP assay in rapid and sensitive detection of *Leishmania* infection.

## Supporting information

S1 Appendix STARD 2015 Checklist for reporting of studies of diagnostic accuracy. (DOCX)

S2 Appendix Flow chart depicting the recruitment and follow up of participants for the validation study of LAMP assay for diagnosis of VL. (DOCX)

S3 Appendix Standard operating protocols provided of LAMP and DBL-LAMP assay. (DOCX)

S4 Appendix Sequence of primers used for LAMP assay. (DOCX)

S5 Appendix Cross-tabulation of the index test results (or their distribution) by the results of the reference standard (rK39 RDT). (DOCX)
