# Supplementary material for: Validation of SYBR green I based closed tube loop mediated isothermal amplification (LAMP) assay and simplified direct-blood-lysis (DBL)-LAMP assay for diagnosis of visceral leishmaniasis (VL)
Source: PLoS Negl Trop Dis. 2018 Nov 15;12(11):e0006922. doi: 10.1371/journal.pntd.0006922 (PMC6264900; doi:10.1371/journal.pntd.0006922)
Supplement: S2 Appendix — (DOCX) [file pntd.0006922.s002.docx]

**Prototypical STARD diagram to report flow of participants through the study.**

Reference standard

rK39 RDT

**N=179**

Reference standard

rK39 RDT

**N=88**

N=88

Excluded

**N=0**

Cases of co-morbidity were excluded.

CZ

Final diagnosis

-Target condition (VL) present (**N=176**)

-Target condition (Non-VL) absent (**N=3**)

-Inconclusive (**N=0**)

N=88

Final diagnosis

-Target condition (VL) present (**N=3**)

-Target condition (Non-VL) absent (**N=85**)

-Inconclusive (**N=0**)

N=88

CZ

Index test

LAMP assay

**N=267**

CZ

CZ

Index test inconclusive

**N=0**

Index test positive

**N=179**

Index test negative

**N=88**

CZ

Eligible participants

**N=267**

CZ

Potentially eligible participants

**N=267**
