# Supplementary material for: Validation of SYBR green I based closed tube loop mediated isothermal amplification (LAMP) assay and simplified direct-blood-lysis (DBL)-LAMP assay for diagnosis of visceral leishmaniasis (VL)
Source: PLoS Negl Trop Dis. 2018 Nov 15;12(11):e0006922. doi: 10.1371/journal.pntd.0006922 (PMC6264900; doi:10.1371/journal.pntd.0006922)
Supplement: S3 Appendix — (DOCX) [file pntd.0006922.s003.docx]

**LAMP ASSAY FOR RAPID AND RELIABLE DIAGNOSIS OF VL**

ICMR-NATIONAL INSTITUTE OF PATHOLOGY, New Delhi

STANDARD OPERATING PROTOCOL

- LAMP with column extracted DNA sample

*IMPORTANT points before starting*

1. Nitrile powder free gloves should be used while setting up LAMP.
2. Recommended to use barrier tips for master mix preparation to prevent any cross contamination.
3. All reagents involved like LAMP mix and *Bst* polymerases should be kept on ice.
4. For ensuring the validity of results it is mandatory to have one positive and negative control.
5. DO NOT open the tubes after DNA amplification. Opening the tube results in a very high risk of DNA contamination to work areas, posing a risk of subsequent false positive LAMP results.

Estimate the concentration of the extracted DNA and adjust it to 50-100ng/µl, use 2 µl DNA of the clinical specimen for putting up the LAMP assay.

PROCEDURE

*Before preparation of master mix ensure that LAMP mix solution is properly thawed and mixed since salts tend to settle at bottom when stored at -30 ˚C.*

1. LAMP master mix should be prepared depending upon the number of samples to be done in the following manner -

| **S.NO.** | **COMPONENTS** | **1.1x** | **11x** |
| --- | --- | --- | --- |
| 1. | LAMP mix | 14.85 µl | 148.5 µl |
| 2. | *Bst* polymerases | 1.1 µl | 11 µl |
| 3. | Nuclease free water | 9.35 µl | 93.5 µl |

b. Mix master mix by giving a brief spin at 2000 rpm for 1 minute. Aliquot 23 µl into 0.2 ml PCR tubes. Add 1 µl of 1:10 diluted SYBR Green I to inner side of the lid of each tube.

c. Add 2 µl of DNA sample and close the tube.

d. Incubate the tubes on dry bath at 65 ˚C for 30 minutes.

e. Let the tubes cool down to RT and then give a brief spin to mix SYBR green I with the amplified product.

f. Check the color change in samples. Positive samples will instantly turn GREEN whereas negatives will remain ORANGE.

**LAMP ASSAY FOR RAPID AND RELIABLE DIAGNOSIS OF VL**

ICMR-NATIONAL INSTITUTE OF PATHOLOGY, New Delhi

STANDARD OPERATING PROTOCOL

- Direct blood Lysis(DBL)-LAMP

*IMPORTANT points before starting*

1. Nitrile powder free gloves should be used while setting up LAMP.
2. Recommended to use barrier tips for master mix preparation to prevent any cross contamination.
3. All reagents involved like LAMP mix and *Bst* polymerases should be kept on ice.
4. For ensuring the validity of results it is mandatory to have one positive and negative control.
5. DO NOT open the tubes after DNA amplification. Opening the tube results in a very high risk of DNA contamination to work areas, posing a risk of subsequent false positive LAMP results.

PROCEDURE

1. Take 50 µl of whole blood and add 50 µl of provided lysis buffer. Mix well and incubate on dry bath at 99 ˚C for 10 minutes.

2. Centrifuge at 13,000 rpm for 5 minutes and collect the supernatant.

*Before preparation of master mix ensure that LAMP mix solution is properly thawed and mixed since salts tend to settle at bottom when stored at -30 ˚C.*

3.LAMP master mix should be prepared depending upon the number of samples to be done in the following manner -

| **S.NO.** | **COMPONENTS** | **1.1x** | **11x** |
| --- | --- | --- | --- |
| 1. | LAMP mix | 14.85 µl | 148.5 µl |
| 2. | *Bst* polymerases | 1.1 µl | 11 µl |
| 3. | Nuclease free water | 6.05 µl | 66.55 µl |

4**.**  Mix master mix by giving a brief spin at 2000 rpm for 1 minute. Aliquot 23 µl into 0.2 ml PCR tubes. Add 1 µl of 1:10 diluted SYBR Green I to inner side of the lid of each tube.

5. Add 5 µl of obtained supernatant and close the tube.

6. Incubate tubes on dry bath at 65 ˚C for 60 minutes.

7. After 60 minutes let the tubes cool down to RT and then give a brief spin to mix SYBR green I with the amplified product.

8. Check the color change in samples. Positive samples will instantly turn GREEN whereas negatives will remain ORANGE.
