# Supplementary material for: Validation of SYBR green I based closed tube loop mediated isothermal amplification (LAMP) assay and simplified direct-blood-lysis (DBL)-LAMP assay for diagnosis of visceral leishmaniasis (VL)
Source: PLoS Negl Trop Dis. 2018 Nov 15;12(11):e0006922. doi: 10.1371/journal.pntd.0006922 (PMC6264900; doi:10.1371/journal.pntd.0006922)
Supplement: S4 Appendix — (DOCX) [file pntd.0006922.s004.docx]

**Primers for LAMP assay**

**Primers for LAMP assay were designed for amplification of Leishmania DNA from *L. donovani* kinetoplast minicircle sequence (Accession no Y11401) using Primer Explorer V4 software. A total of six primers (F3, B3, FIP, BIP, FLP and BLP) were designed.**

***L. donovani* Kinetoplast minicircle DNA, 792 bp**

**>gi | 2910980| emb | Y11401.1|**


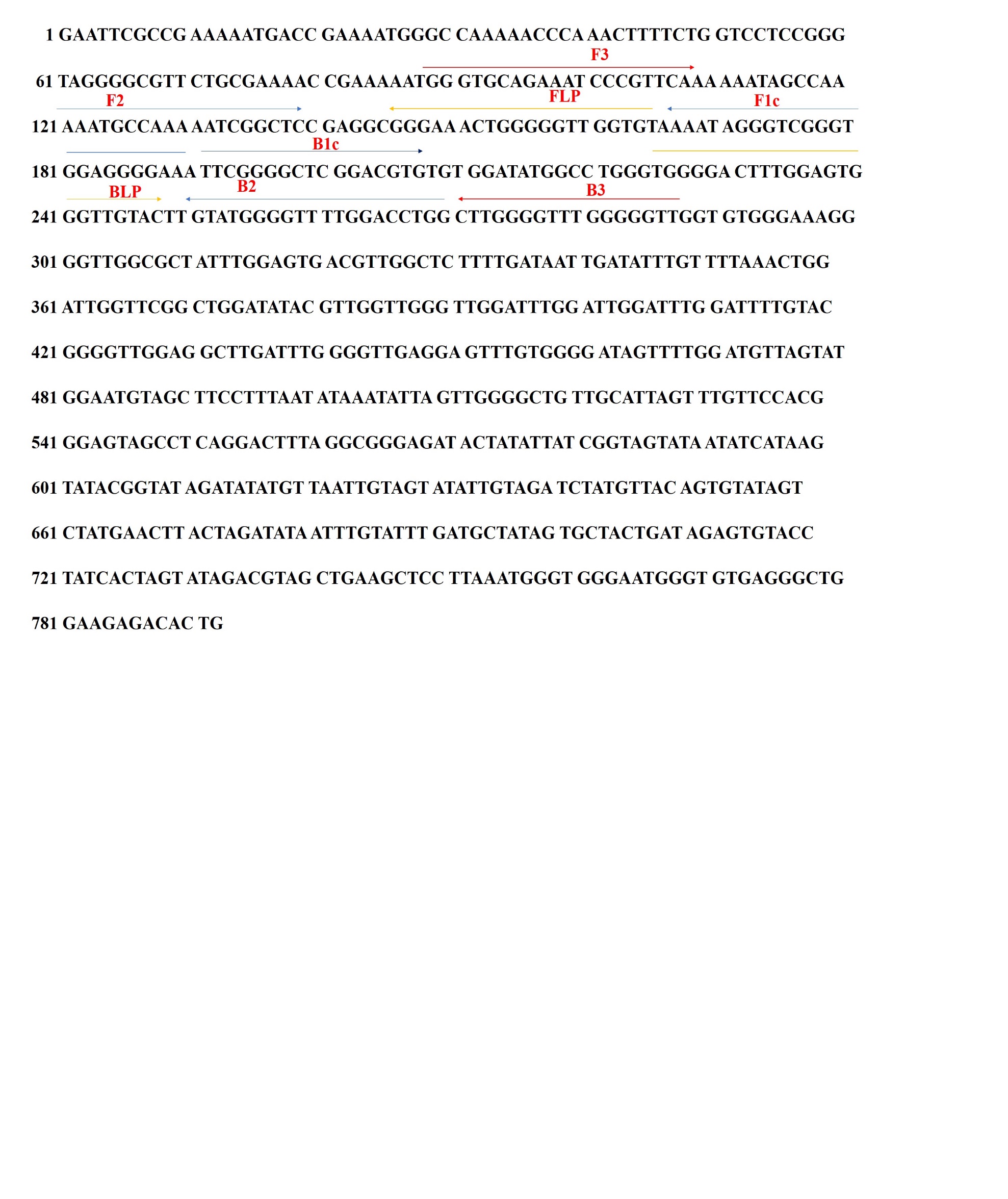


**Primer sequences**

**F3**-5’GGTGCAGAAATCCCGTTCAA3’**, B3-**5’CAACCCCCAAACCCCAAG3’

**Forward Inner Primer (FIP=F1c-F2)** -5’CCCCTCCACCCGACCCTATTT**-**AAATGCCAAAAATCGGCTCC3**’, Backward Inner Primer (BIP=B1c-B2)-**5’TCGGGGCTCGGACGTGTGT-CCAGGTCCAAAACCCCATAC3’

**Forward loop primer (FLP) –** 5’CACCAACCCCCAGTTTCCCG3’**,**

**Backward loop primer (BLP)-** 5’GGGGACTTTGGAGTGGGTTGTA3’
