# Supplementary material for: Validation of SYBR green I based closed tube loop mediated isothermal amplification (LAMP) assay and simplified direct-blood-lysis (DBL)-LAMP assay for diagnosis of visceral leishmaniasis (VL)
Source: PLoS Negl Trop Dis. 2018 Nov 15;12(11):e0006922. doi: 10.1371/journal.pntd.0006922 (PMC6264900; doi:10.1371/journal.pntd.0006922)
Supplement: S5 Appendix — (DOCX) [file pntd.0006922.s005.docx]

**Cross-tabulation of the index test (LAMP assay) results (or their distribution) by the results of the reference standard (rK39 RDT).**

| **Case No.** | **Index Test**  **(LAMP assay)** | **Reference**  **Standard**  **(rK39 RDT)** |  | **Case**  **No.** | **Index Test**  **(LAMP assay)** | **Reference**  **Standard**  **(rK39 RDT)** |
| --- | --- | --- | --- | --- | --- | --- |
|  |  |  |  | 45 | Positive | Positive |
| 1 | Positive | Positive |  | 46 | Positive | Positive |
| 2 | Positive | Positive |  | 47 | Positive | Positive |
| 3 | Positive | Positive |  | 48 | Positive | Positive |
| 4 | Positive | Positive |  | 49 | Positive | Positive |
| 5 | Positive | Positive |  | 50 | Positive | Positive |
| 6 | Positive | Positive |  | 51 | Positive | Positive |
| 7 | Positive | Positive |  | 52 | Positive | Positive |
| 8 | Positive | Positive |  | 53 | Positive | Positive |
| 9 | Positive | Positive |  | 54 | Positive | Positive |
| 10 | Positive | Positive |  | 55 | Positive | Positive |
| 11 | Positive | Positive |  | 56 | Positive | Positive |
| 12 | Positive | Positive |  | 57 | Positive | Positive |
| 13 | Positive | Positive |  | 58 | Positive | Positive |
| 14 | Positive | Positive |  | 59 | Positive | Positive |
| 15 | Positive | Positive |  | 60 | Positive | Positive |
| 16 | Positive | Positive |  | 61 | Positive | Positive |
| 17 | Positive | Positive |  | 62 | Positive | Positive |
| 18 | Positive | Positive |  | 63 | Positive | Positive |
| 19 | Positive | Positive |  | 64 | Positive | Positive |
| 20 | Positive | Positive |  | 65 | Positive | Positive |
| 21 | Positive | Positive |  | 66 | Positive | Positive |
| 22 | Positive | Positive |  | 67 | Positive | Positive |
| 23 | Positive | Positive |  | 68 | Positive | Positive |
| 24 | Positive | Positive |  | 69 | Positive | Positive |
| 25 | Positive | Positive |  | 70 | Positive | Positive |
| 26 | Positive | Positive |  | 71 | Positive | Positive |
| 27 | Positive | Positive |  | 72 | Positive | Positive |
| 28 | Positive | Positive |  | 73 | Positive | Positive |
| 29 | Positive | Positive |  | 74 | Positive | Positive |
| 30 | Positive | Positive |  | 75 | Positive | Positive |
| 31 | Positive | Positive |  | 76 | Positive | Positive |
| 32 | Positive | Positive |  | 77 | Positive | Positive |
| 33 | Positive | Positive |  | 78 | Positive | Positive |
| 34 | Positive | Positive |  | 79 | Positive | Positive |
| 35 | Positive | Positive |  | 80 | Positive | Positive |
| 36 | Positive | Positive |  | 81 | Positive | Positive |
| 37 | Positive | Positive |  | 82 | Positive | Positive |
| 38 | Positive | Positive |  | 83 | Positive | Positive |
| 39 | Positive | Positive |  | 84 | Positive | Positive |
| 40 | Positive | Positive |  | 85 | Positive | Positive |
| 41 | Positive | Positive |  | 86 | Positive | Positive |
| 42 | Positive | Positive |  | 87 | Positive | Positive |
| 43 | Positive | Positive |  | 88 | Positive | Positive |
| 44 | Positive | Positive |  | 89 | Positive | Positive |
| 45 | Positive | Positive |  | 90 | Positive | Positive |
| 46 | Positive | Positive |  | 91 | Positive | Positive |
| 47 | Positive | Positive |  | 92 | Positive | Positive |
| 48 | Positive | Positive |  | 93 | Positive | Positive |
| 49 | Positive | Positive |  | 94 | Positive | Positive |
| 50 | Positive | Positive |  | 95 | Positive | Positive |
| 51 | Positive | Positive |  | 96 | Positive | Positive |
| 52 | Positive | Positive |  | 97 | Positive | Positive |
| 53 | Positive | Positive |  | 98 | Positive | Positive |
| 54 | Positive | Positive |  | 99 | Positive | Positive |
| 55 | Positive | Positive |  | 100 | Positive | Positive |
| 56 | Positive | Positive |  | 101 | Positive | Positive |
| 57 | Positive | Positive |  | 102 | Positive | Positive |
| 58 | Positive | Positive |  | 103 | Positive | Positive |
| 59 | Positive | Positive |  | 104 | Positive | Positive |
| 60 | Positive | Positive |  | 105 | Positive | Positive |
| 61 | Positive | Positive |  | 106 | Positive | Positive |
| 62 | Positive | Positive |  | 107 | Positive | Positive |
| 63 | Positive | Positive |  | 108 | Positive | Positive |
| 64 | Positive | Positive |  | 109 | Positive | Positive |
| 65 | Positive | Positive |  | 110 | Positive | Positive |
| 66 | Positive | Positive |  | 111 | Positive | Positive |
| 67 | Positive | Positive |  | 112 | Positive | Positive |
| 68 | Positive | Positive |  | 113 | Positive | Positive |
| 69 | Positive | Positive |  | 114 | Positive | Positive |
| 70 | Positive | Positive |  | 115 | Positive | Positive |
| 71 | Positive | Positive |  | 116 | Positive | Positive |
| 72 | Positive | Positive |  | 117 | Positive | Positive |
| 73 | Positive | Positive |  | 118 | Positive | Positive |
| 74 | Positive | Positive |  | 119 | Positive | Positive |
| 75 | Positive | Positive |  | 120 | Positive | Positive |
| 76 | Positive | Positive |  | 121 | Positive | Positive |
| 77 | Positive | Positive |  | 122 | Positive | Positive |
| 78 | Positive | Positive |  | 123 | Positive | Positive |
| 79 | Positive | Positive |  | 124 | Positive | Positive |
| 80 | Positive | Positive |  | 125 | Positive | Positive |
| 81 | Positive | Positive |  | 126 | Positive | Positive |
| 82 | Positive | Positive |  | 127 | Positive | Positive |
| 83 | Positive | Positive |  | 128 | Positive | Positive |
| 84 | Positive | Positive |  | 129 | Positive | Positive |
| 85 | Positive | Positive |  | 130 | Positive | Positive |
| 86 | Positive | Positive |  | 131 | Positive | Positive |
| 87 | Positive | Positive |  | 132 | Positive | Positive |
| 88 | Positive | Positive |  | 133 | Positive | Positive |
| 89 | Positive | Positive |  | 134 | Positive | Positive |
| 90 | Positive | Positive |  | 135 | Positive | Positive |
| 91 | Positive | Positive |  | 136 | Positive | Positive |
| 92 | Positive | Positive |  | 137 | Positive | Positive |
| 93 | Positive | Positive |  | 138 | Positive | Positive |
| 94 | Positive | Positive |  | 139 | Positive | Positive |
| 95 | Positive | Positive |  | 140 | Positive | Positive |
| 96 | Positive | Positive |  | 141 | Positive | Positive |
| 97 | Positive | Positive |  | 142 | Positive | Positive |
| 98 | Positive | Positive |  | 143 | Positive | Positive |
| 99 | Positive | Positive |  | 144 | Positive | Positive |
| 100 | Positive | Positive |  | 145 | Positive | Positive |
| 101 | Positive | Positive |  | 146 | Positive | Positive |
| 102 | Positive | Positive |  | 147 | Positive | Positive |
| 103 | Positive | Positive |  | 148 | Positive | Positive |
| 104 | Positive | Positive |  | 149 | Positive | Positive |
| 105 | Positive | Positive |  | 150 | Positive | Positive |
| 106 | Positive | Positive |  | 151 | Positive | Positive |
| 107 | Positive | Positive |  | 152 | Positive | Positive |
| 108 | Positive | Positive |  | 153 | Positive | Positive |
| 109 | Positive | Positive |  | 154 | Positive | Positive |
| 110 | Positive | Positive |  | 155 | Positive | Positive |
| 111 | Positive | Positive |  | 156 | Positive | Positive |
| 112 | Positive | Positive |  | 157 | Positive | Positive |
| 113 | Positive | Positive |  | 158 | Positive | Positive |
| 114 | Positive | Positive |  | 159 | Positive | Positive |
| 115 | Positive | Positive |  | 160 | Positive | Positive |
| 116 | Positive | Positive |  | 161 | Positive | Positive |
| 117 | Positive | Positive |  | 162 | Positive | Positive |
| 118 | Positive | Positive |  | 163 | Positive | Positive |
| 119 | Positive | Positive |  | 164 | Positive | Positive |
| 120 | Positive | Positive |  | 165 | Positive | Positive |
| 121 | Positive | Positive |  | 166 | Positive | Positive |
| 122 | Positive | Positive |  | 167 | Positive | Positive |
| 123 | Positive | Positive |  | 168 | Positive | Positive |
| 124 | Positive | Positive |  | 169 | Positive | Positive |
| 125 | Positive | Positive |  | 170 | Positive | Positive |
| 126 | Positive | Positive |  | 171 | Positive | Positive |
| 127 | Positive | Positive |  | 172 | Positive | Positive |
| 128 | Positive | Positive |  | 173 | Positive | Positive |
| 129 | Positive | Positive |  | 174 | Positive | Positive |
| 130 | Positive | Positive |  | 175 | Positive | Positive |
| 131 | Positive | Positive |  | 176 | Positive | Positive |
| 132 | Positive | Positive |  | 177 | **Negative** | **Positive** |
| 133 | Positive | Positive |  | 178 | **Negative** | **Positive** |
| 134 | Positive | Positive |  | 179 | **Negative** | **Positive** |
| 135 | Positive | Positive |  | 180 | Negative | Negative |
| 136 | Positive | Positive |  | 181 | Negative | Negative |
| 137 | Positive | Positive |  | 182 | Negative | Negative |
| 138 | Positive | Positive |  | 183 | Negative | Negative |
| 139 | Positive | Positive |  | 184 | Negative | Negative |
| 185 | Negative | Negative |  | 233 | Negative | Negative |
| 186 | Negative | Negative |  | 234 | Negative | Negative |
| 187 | Negative | Negative |  | 235 | Negative | Negative |
| 188 | Negative | Negative |  | 236 | Negative | Negative |
| 189 | Negative | Negative |  | 237 | Negative | Negative |
| 190 | Negative | Negative |  | 238 | Negative | Negative |
| 191 | Negative | Negative |  | 239 | Negative | Negative |
| 192 | Negative | Negative |  | 240 | Negative | Negative |
| 193 | Negative | Negative |  | 241 | Negative | Negative |
| 194 | Negative | Negative |  | 242 | Negative | Negative |
| 195 | Negative | Negative |  | 243 | Negative | Negative |
| 196 | Negative | Negative |  | 244 | Negative | Negative |
| 197 | Negative | Negative |  | 245 | Negative | Negative |
| 198 | Negative | Negative |  | 246 | Negative | Negative |
| 199 | Negative | Negative |  | 247 | Negative | Negative |
| 200 | Negative | Negative |  | 248 | Negative | Negative |
| 201 | Negative | Negative |  | 249 | Negative | Negative |
| 202 | Negative | Negative |  | 250 | Negative | Negative |
| 203 | Negative | Negative |  | 251 | Negative | Negative |
| 204 | Negative | Negative |  | 252 | Negative | Negative |
| 205 | Negative | Negative |  | 253 | Negative | Negative |
| 206 | Negative | Negative |  | 254 | Negative | Negative |
| 207 | Negative | Negative |  | 255 | Negative | Negative |
| 208 | Negative | Negative |  | 256 | Negative | Negative |
| 209 | Negative | Negative |  | 257 | Negative | Negative |
| 210 | Negative | Negative |  | 258 | Negative | Negative |
| 211 | Negative | Negative |  | 259 | Negative | Negative |
| 212 | Negative | Negative |  | 260 | Negative | Negative |
| 213 | Negative | Negative |  | 261 | Negative | Negative |
| 214 | Negative | Negative |  | 262 | Negative | Negative |
| 215 | Negative | Negative |  | 263 | Negative | Negative |
| 216 | Negative | Negative |  | 264 | Negative | Negative |
| 217 | Negative | Negative |  | 265 | **Positive** | **Negative** |
| 218 | Negative | Negative |  | 266 | **Positive** | **Negative** |
| 219 | Negative | Negative |  | 267 | **Positive** | **Negative** |
| 220 | Negative | Negative |  |  |  |  |
| 221 | Negative | Negative |  |  |  |  |
| 222 | Negative | Negative |  |  |  |  |
| 223 | Negative | Negative |  |  |  |  |
| 224 | Negative | Negative |  |  |  |  |
| 225 | Negative | Negative |  |  |  |  |
| 226 | Negative | Negative |  |  |  |  |
| 227 | Negative | Negative |  |  |  |  |
